# Significant response to pralsetinib in a medullary thyroid cancer harboring double RET variants of unknown significance

**DOI:** 10.1530/ETJ-22-0044

**Published:** 2022-09-27

**Authors:** Ségolène Hescot, Julien Masliah-Planchon, Pauline du Rusquec, Célia Dupain, Maud Kamal, Vincent Servois, Ivan Bieche

**Affiliations:** 1 Department of Drug Development and Innovation (D3i), Institut Curie, Paris, France; 2 Department of Genetics, Institut Curie, Paris, France; 3 Department of Radiology, Institut Curie, Paris, France

Medullary thyroid carcinoma (MTC) is a rare but aggressive thyroid tumor, with 25% of hereditary and 75% of sporadic forms. *RET* mutations are found in 98% of hereditary MTC and in 55% of sporadic MTC (1). The most frequent somatic *RET* mutation occurs in codon M918, reported in up to 90% of RET-positive MTC cases (2). Selpercatinib and pralsetinib, tyrosine-kinase inhibitors with high specificity for RET protein, recently obtained FDA approval for the treatment of lung and thyroid cancers with *RET* gene mutations or fusions (3, 4). In MTC patients, phase I/II studies with RET inhibitors reported overall response rates of 73% and phase III studies are ongoing (5, 6).

Here, we describe the case of a patient with metastatic MTC who harbored two somatic variants of unknown significance (VUS) in the *RET* gene and responded to pralsetinib.

A 48-year-old male with no previous medical history was referred to our center for liver metastases discovered during cholecystectomy. CT revealed a thyroid tumor, with metastases in the lung, liver, lymph nodes, and bone. MRI showed brain metastases and multiple bone lesions. Liver biopsy was performed and led to the diagnosis of metastatic MTC. His family history was negative, and no germline mutation of the *RET* gene was found. An additional next-generation sequencing (NGS) panel was performed on tumor DNA to detect single-nucleotide variation in 571 genes involved in oncogenesis, as well as copy number variations, tumor mutational burden (TMB), and microsatellite instability status. The tumor showed two VUS of *RET* (NM_020975; c.3047T>C p.(Leu1016Ser), VAF 43%, coverage 3038X) and (NM_020975; c.1770_1820del/p.(Val591_607del) VAF 12%, coverage 3094X). The microsatellite status was stable and the TMB was low with 6.3 variants/Mb. Because of the allele frequency of the missense variant which was not tested in the initial germline mutation, we performed an NGS on patient DNA that confirmed a germline mutation. The disease remained stable between April and September 2020, while a clinical progression with the appearance of symptoms related to hormonal secretion, an increase in calcitonin, and a RECIST progression occurred in December 2020. The case was discussed in our local molecular tumor board, and a treatment with a highly selective RET inhibitor was proposed. The patient was treated with first-line pralsetinib from January 2021 and experienced partial response with a clear clinical and biochemical benefit (Fig. 1 and Table 1). To date, still receiving pralsetinib, he has prolonged disease control for more than a year.

**Figure 1:**
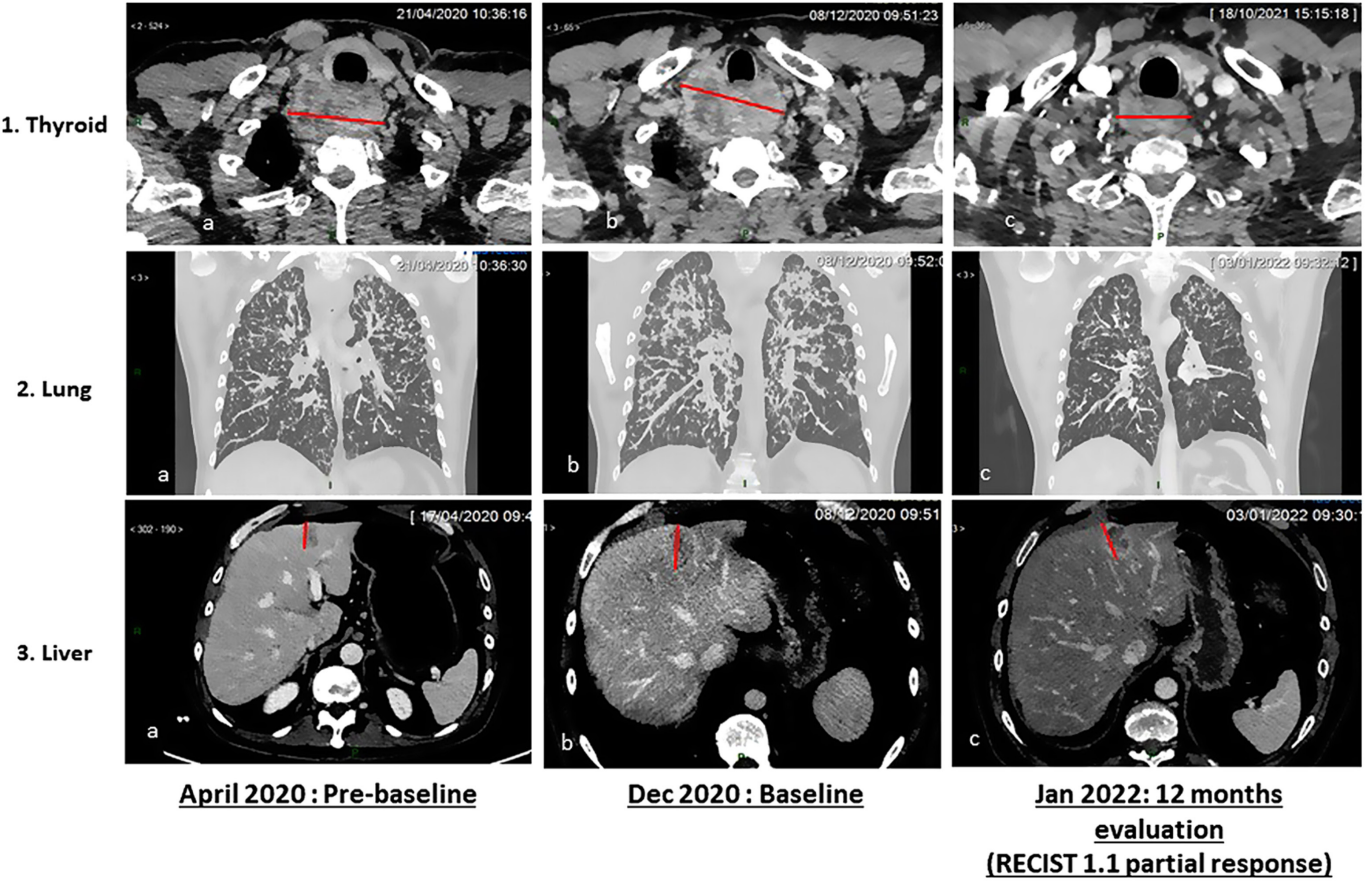
RECIST 1.1 partial response and biochemical response with Pralsetinib. 1. (A, B, and C) Axial CT slices showing a thyroid mass with retrotracheal extension. At 12 months, decrease in size and enhancement of the mass. 2. (A, B, and C) CT scan with coronal reconstruction in maximum intensity projection (MIP) showing diffuse bilateral tiny nodules. The nodules increase in number and size between pre-baseline and baseline examination and decrease significantly at 12 months. 3. (A, B, and C) Axial CT slices showing a liver met in segment III. At 12 months, there is a small reduction in the size of the lesion. However, we note the appearance of a central hypodense zone related to a marked reduction in its vascularization.

**Table 1:** Serial plasma calcitonin concentrations before and during the pralsetinib treatment.

	Calcitonin (ng/L)	CEA (ng/mL)
April 2020: pre-baseline	10,640	858
December 2020: baseline	15,979	1345
January 2022: 12 months evaluation	1617	107

Most of the *RET* somatic mutations occur in codon M918 within exon 16 and in codon C634 within exon 11. The two VUS found in our patient are not reported in population databases or in the medical literature. At a genetic level, *RET* alterations responsible for MTC are missense gain of function mutations located in the extracellular domain of RET (exons 10 or 11) and in the RET tyrosine kinase domain (from exon 13 to exon 19) (7). The p(Leu1016Ser) (c.3047T>C) variant affects the tyrosine kinase domain (exon 19), the difference between Leu and Ser corresponds to a large physicochemical change, the leucine at this position being highly evolutionarily conserved. According to the ACMG criteria, the variant should be classified as a VUS, but cell biology studies are mandatory to explore its role in RET activation. On the other side, Ceccherini *et al.* showed that somatic in-frame deletions do not involve juxtamembranous cysteine residues that strongly activate the RET proto-oncogene (8). The p.Val591_607del (c.1770_1820del) variant found in our patient is very similar (removal of a stretch of amino acids located just upstream the crucial cysteine residue region) and therefore probably pathogenic. The mechanism involved here could be compared to *MET* exon 14 skipping in non-small cell lung cancer which is a primary oncogenic driver very sensitive to selective MET inhibitors (9). In a recent cohort, 13 out of 88 cases presenting *RET* somatic alterations had multiple alterations (2). However, we do not know yet if double mutations that are causative of malignant transformation have a better response to RET inhibitors as already shown for *PIK3CA* or *MET* alterations (10, 11). In our case, it is unclear whether the combination of two variants leads to sensitivity for a RET inhibitor or if only the in-frame deletion is responsible for this effect. At a clinical level, the patient had a metastatic MTC diagnosed at 48 years of age. Somatic *RET*-mutated MTC is known to be more aggressive (2, 12). This case illustrates the value of molecular tumor boards where multidisciplinary discussions can lead to select targeted therapies that benefit patients. Moreover, the tumor response to a highly specific RET inhibitor suggests that this patient’s *RET* indel is causative of MTC.

## Declaration of interest

The authors declare that there is no conflict of interest that could be perceived as prejudicing the impartiality of this case report.

## Funding

This work did not receive any specific grant from any funding agency in the public, commercial, or not-for-profit sector.

## Patient consent

Informed consent has been obtained from the patient for publication of the case report and accompanying images.
